# Supplementary material for: Association between family income to poverty ratio and nocturia in adults aged 20 years and older: A study from NHANES 2005–2010
Source: PLoS One. 2024 May 20;19(5):e0303927. doi: 10.1371/journal.pone.0303927 (PMC11104595; doi:10.1371/journal.pone.0303927)

PLOS ONE

Association between family income to poverty ratio and nocturia status: a study from NHANES 2005-2010

--Manuscript Draft--

**Manuscript Number:**	PONE-D-24-06446
**Article Type:**	Research Article
**Full Title:**	Association between family income to poverty ratio and nocturia status: a study from NHANES 2005-2010
**Short Title:**	Family income to poverty ratio the risk of nocturia
**Corresponding Author:**	Yangtao Jia, master<br>Ningbo Medical Centre Lihuili Hospital<br>Ningbo, CHINA
**Keywords:**	NHANES, nocturia, family income to poverty ratio（PIR）, cross-section study.
**Abstract:**	Abstract<br><br>Background: Nocturia, the most common lower urinary tract symptom (LUTS), significantly impacts socioeconomic factors and individuals' quality of life and is closely related to many diseases. This study utilized data from NHANES 2005-2010 to explore the relationship between family income to poverty ratio (PIR) and the presence of nocturia symptoms in adults aged 20 or older in the United States.<br><br>Methods: Data from the National Health and Nutrition Examination Survey (NHANES) in 2005-2010, including 6,662 adults aged 20 or older, were utilized for this cross-sectional study. The baseline data was used to display the distribution of each characteristic visually. Multiple linear regression and smooth curve fitting were used to study the linear and non-linear correlations between PIR and nocturia. Subgroup analysis and interaction tests were conducted to examine the stability of intergroup relationships.<br><br>Results: Among the 6,662 adult participants aged 20 or older, there were 1,300 households in poverty, 3,671 with moderate income, and 1,691 affluent households. After adjusting for all other covariates, it was found that PIR was significantly negatively correlated with nocturia (OR: 0.875, 95%CI: 0.836-0.916). This trend persisted when PIR was divided into three groups (PIR <1, PIR 1-4, PIR > 4) or quartiles. There was a non-linear negative correlation between PIR and nocturia.<br><br>Conclusion: PIR was highly negatively correlated with nocturia in adults aged 20 or older in the United States. The results of this study were of great significance for the prevention of nocturia.
**Order of Authors:**	Yangtao Jia
	Fangzheng Yang
	Jiacheng Cai
	Huimin Long
	Libin Zhou
	Xinke Dong
**Additional Information:**	
**Question**	**Response**
**Financial Disclosure**<br><br>Enter a financial disclosure statement that describes the sources of funding for the	Yes

Powered by Editorial Manager® and ProduXion Manager® from Aries Systems Corporation

work included in this submission. Review the submission guidelines for detailed requirements. View published research articles from *PLOS ONE* for specific examples.<br><br>This statement is required for submission and **will appear in the published article** if the submission is accepted. Please make sure it is accurate.<br><br>**Funded studies**<br>Enter a statement with the following details:<br>• Initials of the authors who received each award<br>• Grant numbers awarded to each author<br>• The full name of each funder<br>• URL of each funder website<br>• Did the sponsors or funders play any role in the study design, data collection and analysis, decision to publish, or preparation of the manuscript?<br><br>Did you receive funding for this work?	
Please add funding details.<br>as follow-up to "**Financial Disclosure**<br><br>Enter a financial disclosure statement that describes the sources of funding for the work included in this submission. Review the submission guidelines for detailed requirements. View published research articles from *PLOS ONE* for specific examples.<br><br>This statement is required for submission and **will appear in the published article** if the submission is accepted. Please make sure it is accurate.	The authors disclosed receipt of the following financial support for this article: This work was supported by theNaturalScience Foundation of Ningbo Municipality (2021J281), the Key Cultivating Discipline of LihHuiLi Hospital (2022-P09) and Ningbo Key Clinical Speciality Construction Project (2023-BZZ) X

Powered by Editorial Manager® and ProduXion Manager® from Aries Systems Corporation

**Funded studies**<br>Enter a statement with the following details:<br>• Initials of the authors who received each award<br>• Grant numbers awarded to each author<br>• The full name of each funder<br>• URL of each funder website<br>• Did the sponsors or funders play any role in the study design, data collection and analysis, decision to publish, or preparation of the manuscript?<br><br>Did you receive funding for this work?"	
Please select the country of your main research funder (please select carefully as in some cases this is used in fee calculation).<br>as follow-up to "**Financial Disclosure**<br><br>Enter a financial disclosure statement that describes the sources of funding for the work included in this submission. Review the submission guidelines for detailed requirements. View published research articles from *PLOS ONE* for specific examples.<br><br>This statement is required for submission and **will appear in the published article** if the submission is accepted. Please make sure it is accurate.<br><br>**Funded studies**<br>Enter a statement with the following details:<br>• Initials of the authors who received each award<br>• Grant numbers awarded to each author<br>• The full name of each funder<br>• URL of each funder website<br>• Did the sponsors or funders play any role in the study design, data collection and analysis, decision to publish, or preparation of the manuscript?<br><br>Did you receive funding for this work?"	CHINA - CN
**Competing Interests**	The authors have declared that no competing interests exist.

Use the instructions below to enter a competing interest statement for this submission. On behalf of all authors, disclose any competing interests that could be perceived to bias this work—acknowledging all financial support and any other relevant financial or non-financial competing interests.<br><br>This statement is required for submission and **will appear in the published article** if the submission is accepted. Please make sure it is accurate and that any funding sources listed in your Funding Information later in the submission form are also declared in your Financial Disclosure statement.<br><br>View published research articles from *PLOS ONE* for specific examples.<br><br>**NO authors have competing interests**<br><br>Enter: *The authors have declared that no competing interests exist.*<br><br>**Authors with competing interests**<br><br>Enter competing interest details beginning with this statement:<br><br>*I have read the journal's policy and the authors of this manuscript have the following competing interests: [insert competing interests here]*<br><br>* typeset	
**Ethics Statement**<br><br>Enter an ethics statement for this submission. This statement is required if the study involved:<br><br>• Human participants<br>• Human specimens or tissue<br>• Vertebrate animals or cephalopods<br>• Vertebrate embryos or tissues	Ethical consent was not sought since the analysis was conducted on anonymized data sourced from the NHANES database. The participants had already provided informed consent to NHANES when the data were initially published online, thus obviating the need for additional ethical clearance. Numerous prior studies utilizing this dataset have been featured in your journal. This research was endorsed by the National Center for Health Statistics Research Ethics Review Board, with further details available at https://wwwn.cdc.gov/nchs/nhanes.

• Field research<br><br>Write "N/A" if the submission does not require an ethics statement.<br><br>General guidance is provided below. Consult the submission guidelines for detailed instructions. **Make sure that all information entered here is included in the Methods section of the manuscript.**	

Powered by Editorial Manager® and ProduXion Manager® from Aries Systems Corporation

**Format for specific study types**<br><br>**Human Subject Research (involving human participants and/or tissue)**<br>• Give the name of the institutional review board or ethics committee that approved the study<br>• Include the approval number and/or a statement indicating approval of this research<br>• Indicate the form of consent obtained (written/oral) or the reason that consent was not obtained (e.g. the data were analyzed anonymously)<br><br>**Animal Research (involving vertebrate animals, embryos or tissues)**<br>• Provide the name of the Institutional Animal Care and Use Committee (IACUC) or other relevant ethics board that reviewed the study protocol, and indicate whether they approved this research or granted a formal waiver of ethical approval<br>• Include an approval number if one was obtained<br>• If the study involved *non-human primates*, add *additional details* about animal welfare and steps taken to ameliorate suffering<br>• If anesthesia, euthanasia, or any kind of animal sacrifice is part of the study, include briefly which substances and/or methods were applied<br><br>**Field Research**<br><br>Include the following details if this study involves the collection of plant, animal, or other materials from a natural setting:<br>• Field permit number<br>• Name of the institution or relevant body that granted permission	
**Data Availability**<br><br>Authors are required to make all data underlying the findings described fully available, without restriction, and from the time of publication. PLOS allows rare exceptions to address legal and ethical concerns. See the PLOS Data Policy and FAQ for detailed information.	Yes - all data are fully available without restriction

A Data Availability Statement describing where the data can be found is required at submission. Your answers to this question constitute the Data Availability Statement and **will be published in the article**, if accepted.<br><br>**Important:** Stating 'data available on request from the author' is not sufficient. If your data are only available upon request, select 'No' for the first question and explain your exceptional situation in the text box.<br><br>Do the authors confirm that all data underlying the findings described in their manuscript are fully available without restriction?	
**Describe where the data may be found in full sentences. If you are copying our sample text, replace any instances of XXX with the appropriate details.**<br><br>• If the data are **held or will be held in a public repository**, include URLs, accession numbers or DOIs. If this information will only be available after acceptance, indicate this by ticking the box below. For example: *All XXX files are available from the XXX database (accession number(s) XXX, XXX.).*<br>• If the data are all contained **within the manuscript and/or Supporting Information files**, enter the following: *All relevant data are within the manuscript and its Supporting Information files.*<br>• If neither of these applies but you are able to provide **details of access elsewhere**, with or without limitations, please do so. For example:<br><br>*Data cannot be shared publicly because of [XXX]. Data are available from the XXX Institutional Data Access / Ethics Committee (contact via XXX) for researchers who meet the criteria for access to confidential data.*<br><br>*The data underlying the results presented in the study are available from (include the name of the third party*	The dataset analyzed in the present study is accessible on the NHANES website (https://wwwn.cdc.gov/nchs/nhanes).

and contact information or URL).<br>• This text is appropriate if the data are owned by a third party and authors do not have permission to share the data.<br><br>* typeset	
Additional data availability information:	Tick here if your circumstances are not covered by the questions above and you need the journal’s help to make your data available.

Powered by Editorial Manager® and ProduXion Manager® from Aries Systems Corporation

Manuscript Click here to access/download;Manuscript;Manuscript.docx

Association between family income to poverty ratio and nocturia status: a study from NHANES 2005-2010

Author list: Yangtao Jia[1¶], Jiacheng Cai[1¶], Fangzheng Yang[1&], Xinke Dong[1&], Huimin Long[1*], Libin Zhou[2*]

The Affiliated Lihuili Hospital of Ningbo University, Ningbo, Zhejiang, 315040, People's Republic of China

* Corresponding author

1* Huimin Long1*, the Affiliated Lihuili Hospital of Ningbo University, Ningbo, Zhejiang, 315040, People's Republic of China,

Email: longhuiming@vip.sina.com

2* Libin Zhou2*, the Affiliated Lihuili Hospital of Ningbo University, Ningbo, Zhejiang, 315040, People's Republic of China,

Email: zlburo2013@sina.com

¶ These authors contributed equally to this work

& These authors also contributed equally to this work.

Abstract

Background: Nocturia, the most common lower urinary tract symptom (LUTS), significantly impacts socioeconomic factors and individuals' quality of life and is closely related to many diseases. This study utilized data from NHANES 2005-2010 to explore the relationship between family income to poverty ratio (PIR) and the presence of nocturia symptoms in adults aged 20 or older in the United States.

Methods: Data from the National Health and Nutrition Examination Survey (NHANES) in 2005-2010, including 6,662 adults aged 20 or older, were utilized for this cross-sectional study. The baseline data was used to display the distribution of each characteristic visually. Multiple linear regression and smooth curve fitting were used to study the linear and non-linear correlations between PIR and nocturia. Subgroup analysis and interaction tests were conducted to examine the stability of intergroup relationships.

Include the number and proportion of the sample with nocturia

Results: Among the 6,662 adult participants aged 20 or older, there were 1,300 households in poverty, 3,671 with moderate income, and 1,691 affluent households. After adjusting for all other covariates, it was found that PIR was significantly negatively correlated with nocturia (OR: 0.875, 95%CI: 0.836-0.916). This trend persisted when PIR was divided into three groups (PIR <1, PIR 1-4, PIR > 4) or quartiles. There was a non-linear negative correlation between PIR and nocturia. Include the test statistics

The conclusion of the abstract could be improved by making it more concise and focused on the key findings and implications of the study.

Conclusion: PIR was highly negatively correlated with nocturia in adults aged 20 or older in the United States. The results of this study were of great significance for the prevention of nocturia.

Suggestion for the conclusion
This study found that Lower PIR was associated with a higher risk of nocturia in adults aged 20 or older in the United States. These findings highlight the importance of considering socioeconomic factors in the prevention and management of nocturia, and underscore the need for further research to understand the underlying mechanisms and develop targeted interventions.

Introduction

The International Continence Society (ICS) defined nocturia as the condition in which an individual wakes one or more times to void at night(1). Clinically speaking, nocturia was typically deemed clinically significant if it happened two or more times nightly. Prior studies had associated nocturia occurring more than twice per night with a decline in quality of life.(2, 3). Nocturia is the most prevalent and bothersome among all Lower Urinary Tract Symptoms (LUTS), with as many as 60% of elderly individuals (>70 years) and 15-20% of young adults (20-40 years) experiencing voiding two or more times at night(1, 4). Furthermore, nocturia was associated not only with benign prostatic hyperplasia (BPH)(5), obstructive sleep apnea (OSA)(6), coronary artery disease(7), diabetes(8), sleep disorders(6, 9) and other conditions, but also with increased all-cause mortality(3). Therefore, decreasing nocturia could have profound positive effects on quality of life.

The ratio of family income to poverty (PIR) is calculated by dividing family (or individual) income by the poverty threshold specific to each survey year and is an effective measure

of income disparity closely related to human life and health(10). Previous studies showed that lower family income led to poorer health protection, including the inability to access timely and effective preventive and diagnostic measures, which might have led to various diseases. (11). Furthermore, some claimed that PIR was associated with urgent urinary incontinence in adults. As PIR increased, the risk of urinary incontinence significantly decreased(12). Additionally, research has found that children from lower-income families are more likely to experience bed-wetting at night(13). Because large-scale cross-sectional studies investigating the relationship between nocturia and PIR levels were scarce, we utilized data from NHANES 2005-2010 to explore a large population of adults aged 20 and above in the United States.

Materials and Methods

2.1 Data source

The National Health and Nutrition Examination Survey (NHANES) is a biennial population-based cross-sectional study in the United States that gathers nationally representative data on civilians. This study received approval from the National Center for Health Statistics (NCHS) Research Ethics Review Board, with all participants providing written informed consent. Our analysis focused on participants aged ≥ 20 from three NHANES cycles spanning 2005 to 2010. Data utilized in our study are accessible through the NHANES website (https://www.cdc.gov/nchs/nhanes/index.htm).

2.2 Study population and variables

In this survey, we included 31,034 participants from 2005 to 2010, excluding those under 20 years old (n = 13,902), with missing nocturnal voiding data (n = 2,296), missing Poverty Income Ratio (PIR) data (n = 1,087), or meeting specific criteria indicating potential bias (n = 316). Additionally, 7,149 participants had missing data on other covariates. The final sample consisted of 6,662 participants. The participant selection process is outlined in Figure 1.

Figure 1 Flow chart illustrating the selection of participants in NHANES from 2005 to 2010

The PIR was the independent variable, with nocturia as the dependent variable. Based on family income and federally recognized poverty levels, PIR was categorized into three groups: poor (PIR <1), middle class (PIR 1-4), and affluent (PIR > 4) (14). Nocturia was defined as answering "two times" or "two times or more" to a standardized questionnaire item regarding nocturnal voiding frequency.

The rationale for the categorization of PIR into three groups (PIR <1, PIR 1-4, PIR > 4) should be better explained and supported by relevant literature or guidelines

We explored several variables routinely collected in NHANES, including age, gender, race, education level, marital status, drinking status, BMI, and serum cotinine concentration. Hypertension and diabetes were defined based on standardized questionnaire responses. Hypertension was determined by systolic blood pressure levels ≥ 130 mm Hg and/or diastolic blood pressure levels ≥ 80 mm Hg or answer "yes" to any

of the following questions: "Have you ever been told by a doctor or health professional that you had diabetes or sugar diabetes?" "Because of high blood pressure/hypertension, have you ever been told to take prescribed medicine?"(15). Diabetes was defined by glycosylated hemoglobin levels ≥ 6.5% and/or fasting blood glucose levels ≥ 126 mg/dl, or answer "yes" to any of the following questions: "Have you ever been told by a doctor or health professional that you had diabetes or sugar diabetes?" "Are you now taking diabetic pills to lower your blood sugar?"(16).

2.3 Statistical analysis

Baseline characteristics were summarized for continuous and categorical variables, categorized by three levels of PIR. Continuous variables were presented as mean ± standard deviation, while categorical variables were presented as frequencies and percentages. We employed multivariate logistic regression models to examine the linear relationship between nocturia and PIR, utilizing a weighted smooth curve fitting method to assess non-linear associations. Odds ratios (OR) and corresponding 95% confidence intervals (CI) quantified the strength of association. Model 1 remained unadjusted, while Model 2 adjusted for age, gender, and race/ethnicity. Model 3 additionally adjusted for marital status, education level, serum cotinine concentration, alcohol consumption status, diabetes status, hypertension status, and BMI status. Interaction and stratified analyses were conducted considering the listed covariates. Statistical analyses were performed using STATA v16.0 and R (version 4.2.2). A significance level of $p < 0.05$ was applied.

Explain the rationale for using three models.

Results

3.1 Baseline characteristics

We included 6,662 participants from NHANES cycles between 2005 and 2010 and divided the weighted features into three groups based on PIR. (Table 1) The mean age of the participants was 48.35 ± 0.23, with an average PIR of 3.04 ± 024. Compared to low-income individuals (PIR < 1), those with high income (PIR ≥4) were older (PIR < 1: 43.26 years vs PIR ≥4: 48.93 years) and predominantly male (PIR < 1: 43.92% vs PIR ≥4: 53.40%). They were also more likely to be Non-Hispanic White (PIR < 1: 36.23% vs PIR ≥4: 64.64%) and have completed high school or above (PIR < 1: 24.46% vs PIR ≥4: 75.52%). Furthermore, they were more inclined to be married/living with partner (PIR < 1: 47.69% vs PIR ≥4: 74.93%). The number of individuals with serum cotinine concentration >3 was often lower (PIR < 1: 41.85% vs PIR ≥4: 19.04%), while the number of those consuming alcohol more than 12 times a year was higher (PIR < 1: 65.46% vs PIR ≥4: 79.72%).

Table 1 Baseline characteristics of participants stratified by PIR. Ensure uniformity in the fonts

PIR	<1	1-4	>4	total	P value
**N**	1300	3671	1691	6662	
**PIR**	0.62±0.01	2.36±0.18	4.84±0.01	3.04±0.24	0.222
**Age (years)**	43.26±0.54	49.18±0.34	48.93±0.36	48.35±0.23	0.077
**Nocturia**					<0.0001
No	611(47%)	1,915(52.17%)	997(58.96%)	3,523(52.88%)	

Yes	689(53%)	1,756(47.83%)	694(41.04%)	3,139(47.12%)	
**Gender**					<0.0001
Male	571(43.92%)	1,803(49.11%)	903(53.4%)	3,277(49.19%)	
Female	729(56.08%)	1,868(50.89%)	788(46.6%)	3,385(50.81%)	
**Age**					<0.0001
20-34	377(29%)	825(22.47%)	292(17.27%)	1,494(22.43%)	
35-49	332(25.54%)	820(22.34%)	438(25.90%)	1,590(23.87%)	
50-64	326(25.08%)	873(23.78%)	607(35.90%)	1,806(27.11%)	
≥65	265(20.38%)	1,153(31.41%)	354(20.93%)	1,772(26.60%)	
**Race**					<0.0001
Mexican American	355(27.31%)	716(19.5%)	153(9.05%)	1,224(18.37%)	
Other Hispanic	141(10.85%)	295(8.04%)	101(5.97%)	537(8.06%)	
Non-Hispanic White	471(36.23%)	1,740(47.4%)	1,093(64.64%)	3,304(49.59%)	
Non-Hispanic Black	281(21.62%)	779(21.22%)	261(15.43%)	1,321(19.83%)	
Other Race	52(4%)	141(3.84%)	83(4.91%)	276(4.14%)	
**Education level**					<0.0001
Less than high school	697(53.62%)	1,117(30.43%)	140(8.28%)	1,954(29.33%)	
High school	285(21.92%)	1,031(28.08%)	274(16.2%)	1,590(23.87%)	
More than high school	318(24.46%)	1,523(41.49%)	1,277(75.52%)	3,118(46.8%)	
**Marital status**					<0.0001
Widowed/Divorced/Separated/Never married	680(52.31%)	1,464(39.88%)	424(25.07%)	2,568(38.55%)	
Married/Living with partner	620(47.69%)	2,207(60.12%)	1,267(74.93%)	4,094(61.45%)	
**Cotinine**					<0.0001
<0.015	164(12.62%)	718(19.56%)	422(24.96%)	1,304(19.57%)	
0.015-3	592(45.54%)	2,024(55.13%)	947(56%)	3,563(53.48%)	
>3	544(41.85%)	929(25.31%)	322(19.04%)	1,795(26.94%)	
**BMI**					0.024
<25	343(26.38%)	932(25.39%)	472(27.91%)	1,747(26.22%)	
25-30	422(32.46%)	1,209(32.93%)	593(35.07%)	2,224(33.38%)	
≥30	535(41.15%)	1,530(41.68%)	626(37.02%)	2,691(40.39%)	

**Drink at least 12 drinks/year**					<0.0001
No	449(34.54%)	1,210(32.96%)	343(20.28%)	2,002(30.05%)	
Yes	851(65.46%)	2,461(67.04%)	1,348(79.72%)	4,660(69.95%)	
**Hypertension history**					0.005
No	533(41%)	1,372(37.37%)	702(41.51%)	2,607(39.13%)	
Yes	767(59%)	2,299(62.63%)	989(58.49%)	4,055(60.87%)	
**Diabetes history**					<0.0001
No	901(69.31%)	2,544(69.3%)	1,321(78.12%)	4,766(71.54%)	
Yes	399(30.69%)	1,127(30.7%)	370(21.88%)	1,896(28.46%)	

3.2 Relationship between PIR and Nocturia

The results of multivariate linear regression analysis in three models indicated a significant negative correlation between PIR and nocturia (Model 1, OR = 0.868, 95% CI: 0.836-0.900; Model 2, OR = 0.862, 95% CI: 0.828-0.897; Model 3, OR = 0.875, 95% CI: 0.836-0.916). Further analysis treating PIR as a categorical variable, with poor individuals (PIR<1) as reference, revealed a significant reduction in the risk of nocturia for the middle class (PIR 1-4) and affluent individuals (PIR>4). When PIR was divided into quartiles and compared with Q1, a significant negative correlation with nocturia risk was observed in Q2, Q3, and Q4. (Table 2) Additionally, we conducted restricted cubic spline regression analysis in Model 3, indicating a non-linear negative correlation between PIR and nocturia.(Figure 2)

Table 2 Odds ratios for the prevalence of nocturia stratified by PIR.

	Model 1	P value	Model 2	P value	Model 3	P value

PIR	0.868(0.836-0.900)	<0.0001	0.862(0.828-0.897)	<0.0001	0.875（0.836-0.916）	<0.0001
PIR						
<1	Reference		Reference		Reference	
1-4	0.81(0.716,0.923)	0.001	0.722(0.632,0.825)	<0.0001	0.738(0.642,0.848)	<0.0001
>4	0.617(0.534-0.714)	<0.0001	0.572(0.490,0.669)	<0.0001	0.627(0.527,0.745)	<0.0001
PIR						
Q1	Reference		Reference		Reference	
Q2	0.830(0.704-0.978)	0.026	0.726(0.612-0.862)	<0.0001	0.724(0.607-0.865)	<0.0001
Q3	0.627(0.531-0.740)	<0.0001	0.579(0.486-0.690)	<0.0001	0.591(0.489-0.713)	<0.0001
Q4	0.564(0.479-0.663)	<0.0001	0.528(0.443-0.628)	<0.0001	0.564(0.463-0.686)	<0.0001

Model 1 adjusts for none.

Model 2 adjusts for age, ethnicity and gender.

Model 3 adjusts for age, gender, ethnicity, education, BMI, marital status, cotinine, alcohol status, Hypertension history and Diabetes history.

Q1: PIR≤1.15, Q2:1.15≤PIR<2.14, Q3: 2.14≤PIR<4.08, Q4: PIR>4.08.

Figure 2 Restricted cubic splines showing the relationship between PIR and the presence of nocturia.

3.3 Subgroup analysis

Based on all covariates, we found an unstable correlation between PIR and nocturia in the stratified subgroup analysis. Although a negative correlation between PIR and nocturia was observed in the majority of subgroups, the results of interaction tests provided no evidence of an association between this negative correlation and the variables.(Figure 3)

Figure 3 Subgroup analysis of the association between family PIR and nocturia status

Discussion

This study investigated the relationship between PIR and nocturia symptoms in individuals aged 20 and above. Our model construction and analysis revealed a significant negative correlation between PIR and nocturia symptoms (PR: 0.875; 95% CI: 0.836-0.91; P < 0.0001). Furthermore, constructing restricted cubic spline (RCS) curves unveiled a notable non-linear relationship, indicating a gradual reduction in the risk of nocturia with increasing PIR (P value < 0.0001, P for non-linear = 0.0093). Although subgroup analyses were conducted, interaction results indicated that various covariates were not significantly dependent on the relationship between PIR and nocturia.

Nocturia was a prevalent chronic condition associated with various ailments, such as cardiovascular diseases, endocrine disorders, chronic respiratory diseases, and mortality, frequently leading to a considerable decline in quality of life (17-21). Nevertheless, existing treatment and prevention methods were limited, yielding unsatisfactory management outcomes for nocturia (1). Prior studies identified several nocturia-related factors, including reduced bladder volume, sleep disturbances, and nocturnal polyuria (22-24). Nevertheless, as research on nocturia advanced, some studies revealed a significant association between economic income and nocturia. Consequently, we conducted a study utilizing the NHANES database to investigate the relationship between PIR and nocturia. Our results aligned with prior findings suggesting that lower household

income was often linked with more adverse effects, and there existed an inverse correlation between PIR levels and nocturia. For instance, a recent cross-sectional study conducted among the Danish population corroborated our findings, indicating a significant decrease in the risk of nocturia among individuals with higher household incomes(25). Additionally, previous studies had underscored obesity, hypertension, and diabetes as pivotal risk factors for nocturia, aligning with our findings(8, 26, 27). Based on these findings, we hypothesized that PIR may indicate the overall condition of nocturia, offering guidance for its diagnosis and treatment in clinical practice.

Although the precise mechanism by which PIR influences nocturia remained elusive, we offered potential explanations grounded in existing research. Epidemiological studies indicated that low-income families faced environments detrimental to physical health, struggled with healthy food affordability, and lacked adequate medical coverage(28, 29). Additionally, lower family income correlated with poorer family health protection, hindering timely disease prevention, diagnosis and treatment, thus fostering disease occurrence and progression(11, 30). For instance, women with lower family incomes discussed urinary incontinence with doctors less frequently compared to their higher-income counterparts(31). Conversely, individuals with higher family incomes could promptly intervene in diseases through early-stage examinations, inhibiting disease progression. (32). Furthermore, individuals of lower socioeconomic status often contended with heightened stress hormone levels due to various social and psychological factors. (33, 34). Stress, as shown in previous studies, played a significant

role in the pathophysiology of overactive bladder (OAB) and urge urinary incontinence (UUI), both impacting urinations. (34, 35). Considering these points, we asserted that the influence of income level on nocturia risk was plausible, reinforcing our experimental results.

A significant strength of our study lies in establishing a nationally representative cohort, bolstering the generalizability of our findings. Additionally, this analysis marked the first attempt to explore the relationship between nocturia and PIR based on the NHANES database. Furthermore, our study revealed a negative correlation between PIR and nocturia, holding implications for clinical diagnosis. Nonetheless, being a cross-sectional analysis, causal relationships and potential mechanisms of influence could not be discussed. Moreover, inaccuracies in the included covariates may have impacted result precision. Lastly, the directionality of causality in this association remained indeterminate, the possibility of increased nocturia risk leading to a decrease in household income could not be ruled out.

Conclusion

We have established the correlation between PIR and the incidence of nocturia. An elevation in PIR correlates with a reduced likelihood of experiencing nocturia, indicating its potential utility as an indicator for predicting the probability of developing nocturia. Despite offering specific clinical insights, the precise underlying mechanisms remain

elusive. Therefore, further randomized controlled trials are warranted to furnish additional evidence for preventing and screening nocturia within the populace.

Acknowledgments

We extend our gratitude to all participants in the US National Health and Nutrition Examination Survey (NHANES) and value the unrestricted access offered to the public by the NHANES database.

References

1. Dani H, Esdaille A, Weiss JP. Nocturia: aetiology and treatment in adults. *Nat Rev Urol* 2016:13:573-83.doi:10.1038/nrurol.2016.134

2. Bryan RT, Zeegers MP. Editorial comment on: Nocturia frequency, bother, and quality of life: how often is too often? A population-based study in Finland. *Eur Urol* 2010:57:496-7.doi:10.1016/j.eururo.2009.03.081

3. Kupelian V, Fitzgerald MP, Kaplan SA, Norgaard JP, Chiu GR, Rosen RC. Association of nocturia and mortality: results from the Third National Health and Nutrition Examination Survey. *J Urol* 2011:185:571-7.doi:10.1016/j.juro.2010.09.108

4. Bosch JL, Weiss JP. The prevalence and causes of nocturia. *J Urol* 2013:189:S86-92.doi:10.1016/j.juro.2012.11.033

5. Chughtai B, Forde JC, Thomas DD, Laor L, Hossack T, Woo HH, et al. Benign prostatic

hyperplasia. *Nat Rev Dis Primers* 2016:2:16031.doi:10.1038/nrdp.2016.31

6. Papworth E, Dawson S, Henderson EJ, Eriksson SH, Selsick H, Rees J, et al. Association of Sleep Disorders with Nocturia: A Systematic Review and Nominal Group Technique Consensus on Primary Care Assessment and Treatment. *Eur Urol Focus* 2022:8:42-51.doi:10.1016/j.euf.2021.12.011

7. Khan SA, Rahman EU, Chobufo MD, Yasin A, Monaghan TF, Weiss JP, et al. Nocturia is an Independent Predictor of Abdominal Aortic Calcification in Women: Results from the National Health and Nutrition Examination Survey. *J Community Health* 2020:45:1111-5.doi:10.1007/s10900-020-00893-x

8. Fu Z, Wang F, Dang X, Zhou T. The association between diabetes and nocturia: A systematic review and meta-analysis. *Front Public Health* 2022:10:924488.doi:10.3389/fpubh.2022.924488

9. Ancoli-Israel S, Bliwise DL, Norgaard JP. The effect of nocturia on sleep. *Sleep Med Rev* 2011:15:91-7.doi:10.1016/j.smrv.2010.03.002

10. Minhas AMK, Jain V, Li M, Ariss RW, Fudim M, Michos ED, et al. Family income and cardiovascular disease risk in American adults. *Sci Rep* 2023:13:279.doi:10.1038/s41598-023-27474-x

11. Ward E, Halpern M, Schrag N, Cokkinides V, DeSantis C, Bandi P, et al. Association of insurance with cancer care utilization and outcomes. *CA Cancer J Clin* 2008:58:9-31.doi:10.3322/CA.2007.0011

12. Lee JA, Johns TS, Melamed ML, Tellechea L, Laudano M, Stern JM, et al. Associations between Socioeconomic Status and Urge Urinary Incontinence: An Analysis of NHANES

2005 to 2016. *J Urol* 2020:203:379-84.doi:10.1097/JU.0000000000000542

13. Barone JG, Ramasamy R, Farkas A, Lerner E, Creenan E, Salmon D, et al. Breastfeeding during infancy may protect against bed-wetting during childhood. *Pediatrics* 2006:118:254-9.doi:10.1542/peds.2005-2738

14. Zhao Y, Zhao J, Xie R, Zhang Y, Xu Y, Mao J, et al. Association between family income to poverty ratio and HPV infection status among U.S. women aged 20 years and older: a study from NHANES 2003-2016. *Front Oncol* 2023:13:1265356.doi:10.3389/fonc.2023.1265356

15. Whelton PK, Carey RM, Aronow WS, Casey DE, Jr., Collins KJ, Dennison Himmelfarb C, et al. 2017 ACC/AHA/AAPA/ABC/ACPM/AGS/APhA/ASH/ASPC/NMA/PCNA Guideline for the Prevention, Detection, Evaluation, and Management of High Blood Pressure in Adults: Executive Summary: A Report of the American College of Cardiology/American Heart Association Task Force on Clinical Practice Guidelines. *Circulation* 2018:138:e426-e83.doi:10.1161/CIR.0000000000000597

16. ElSayed NA, Aleppo G, Aroda VR, Bannuru RR, Brown FM, Bruemmer D, et al. 2. Classification and Diagnosis of Diabetes: Standards of Care in Diabetes-2023. *Diabetes Care* 2023:46:S19-S40.doi:10.2337/dc23-S002

17. Miranda Ede P, Gomes CM, Torricelli FC, de Bessa JJ, de Castro JE, Ferreira BR, et al. Nocturia is the Lower Urinary Tract Symptom With Greatest Impact on Quality of Life of Men From a Community Setting. *Int Neurourol J* 2014:18:86-90.doi:10.5213/inj.2014.18.2.86

18. Agarwal A, Eryuzlu LN, Cartwright R, Thorlund K, Tammela TL, Guyatt GH, et al. What is the most bothersome lower urinary tract symptom? Individual- and population-level

perspectives for both men and women. *Eur Urol* 2014:65:1211-7.doi:10.1016/j.eururo.2014.01.019

19. Moon S, Chung HS, Yu JM, Ko KJ, Choi DK, Kwon O, et al. The Association Between Obesity and the Nocturia in the U.S. Population. *Int Neurourol J* 2019:23:169-76.doi:10.5213/inj.1938062.031

20. Bliwise DL, Howard LE, Moreira DM, Andriole GL, Hopp ML, Freedland SJ. Nocturia and associated mortality: observational data from the REDUCE trial. *Prostate Cancer Prostatic Dis* 2019:22:77-83.doi:10.1038/s41391-018-0090-5

21. Funada S, Tabara Y, Setoh K, Negoro H, Akamatsu S, Yoshino T, et al. Impact of Nocturia on Mortality: The Nagahama Study. *J Urol* 2020:204:996-1002.doi:10.1097/JU.0000000000001138

22. Presicce F, Puccini F, De Nunzio C, Melchionna A, Lombardo R, Tubaro A. Variations of Nighttime and Daytime Bladder Capacity in Patients with Nocturia: Implication for Diagnosis and Treatment. *J Urol* 2019:201:962-6.doi:10.1097/JU.0000000000000022

23. Endeshaw YW, Johnson TM, Kutner MH, Ouslander JG, Bliwise DL. Sleep-disordered breathing and nocturia in older adults. *J Am Geriatr Soc* 2004:52:957-60.doi:10.1111/j.1532-5415.2004.52264.x

24. Song J, Ke B, Fang X. Association of nocturia of self-report with estimated glomerular filtration rate: a cross-sectional study from the NHANES 2005-2018. *Sci Rep* 2023:13:13924.doi:10.1038/s41598-023-39448-0

25. Rubach A, Balasubramaniam K, Storsveen MM, Elnegaard S, Jarbol DE. Healthcare-seeking with bothersome lower urinary tract symptoms among men in the Danish population:

the impact of lifestyle and socioeconomic status. *Scand J Prim Health Care* 2019:37:155-64.doi:10.1080/02813432.2019.1608412

26. Chen J, Liu Z, Yang L, Zhou J, Ma K, Peng Z, et al. Relationship between nocturia and hypertension: findings from the NHANES 2005-2016. *Front Cardiovasc Med* 2023:10:1165092.doi:10.3389/fcvm.2023.1165092

27. Bjorge T, Tretli S, Engeland A. Relation of height and body mass index to renal cell carcinoma in two million Norwegian men and women. *Am J Epidemiol* 2004:160:1168-76.doi:10.1093/aje/

28. Xie R, Liu Y, Wang J, Zhang C, Xiao M, Liu M, et al. Race and Gender Differences in the Associations Between Cadmium Exposure and Bone Mineral Density in US Adults. *Biol Trace Elem Res* 2023:201:4254-61.doi:10.1007/s12011-022-03521-y

29. Seabrook JA, Avison WR. Socioeconomic status and cumulative disadvantage processes across the life course: implications for health outcomes. *Can Rev Sociol* 2012:49:50-68.doi:10.1111/j.1755-618x.2011.01280.x

30. Curtis LJ. An economic perspective on the causal explanations for the socioeconomic inequalities in health. *Rev Panam Salud Publica* 2018:42:e53.doi:10.26633/RPSP.2018.53

31. Duralde ER, Walter LC, Van Den Eeden SK, Nakagawa S, Subak LL, Brown JS, et al. Bridging the gap: determinants of undiagnosed or untreated urinary incontinence in women. *Am J Obstet Gynecol* 2016:214:266 e1- e9.doi:10.1016/j.ajog.2015.08.072

32. Bor J, Cohen GH, Galea S. Population health in an era of rising income inequality: USA, 1980-2015. *Lancet* 2017:389:1475-90.doi:10.1016/S0140-6736(17)30571-8

33. Cohen S, Doyle WJ, Baum A. Socioeconomic status is associated with stress hormones.

Psychosom Med 2006:68:414-20.doi:10.1097/01.psy.0000221236.37158.b9

34. Wrobel A, Serefko A, Poleszak E, Rechberger T. Fourteen-day administration of corticosterone may induce detrusor overactivity symptoms. *Int Urogynecol J* 2016:27:1713-21.doi:10.1007/s00192-016-3027-3

35. Smith AL, Hantsoo L, Malykhina AP, File DW, Valentino R, Wein AJ, et al. Basal and stress-activated hypothalamic pituitary adrenal axis function in postmenopausal women with overactive bladder. *Int Urogynecol J* 2016:27:1383-91.doi:10.1007/s00192-016-2988-6

Figure 1

Click here to access/download;Figure;Figure 1.tiff

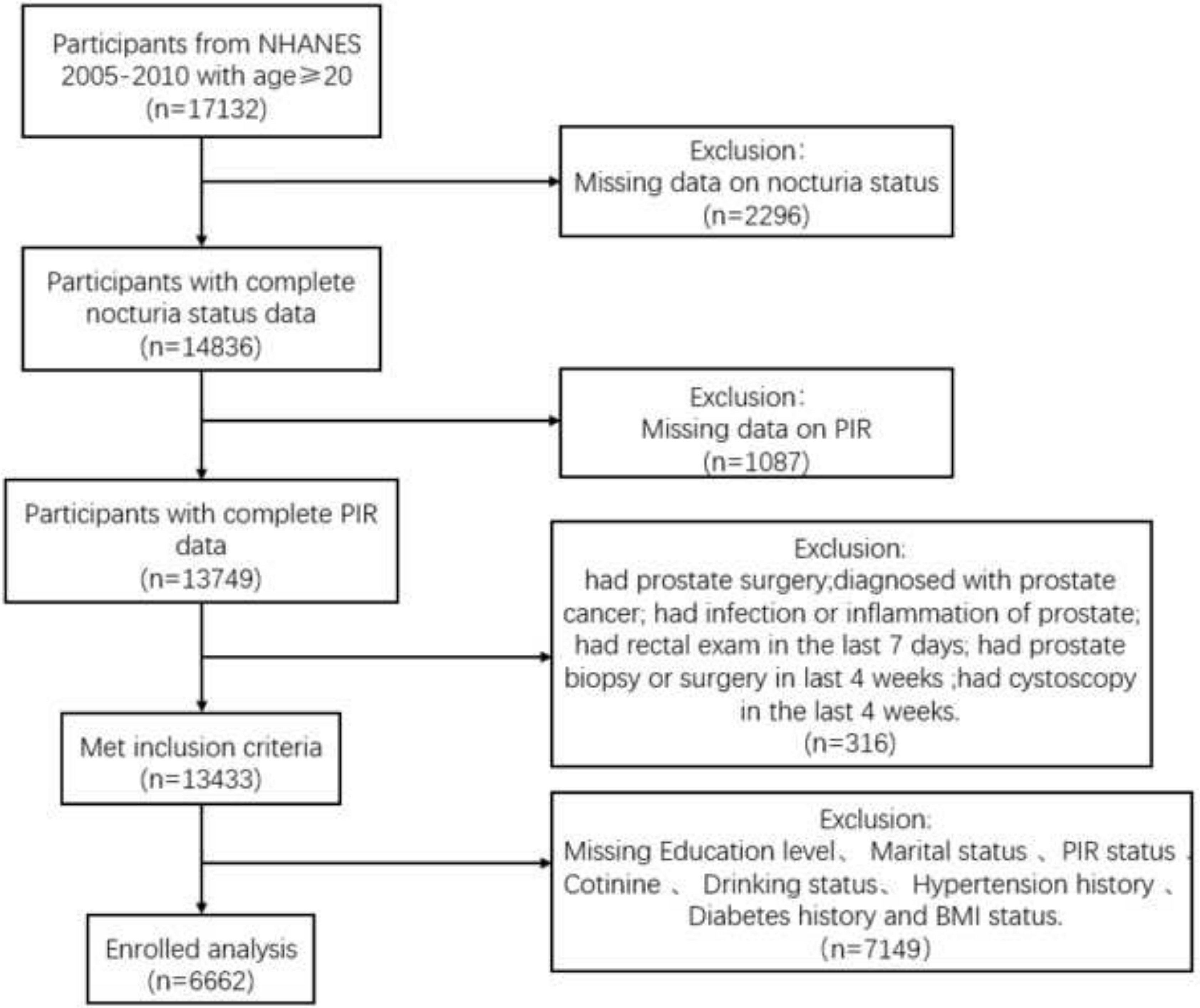

Figure 2

Click here to access/download;Figure;Figure 2.tiff

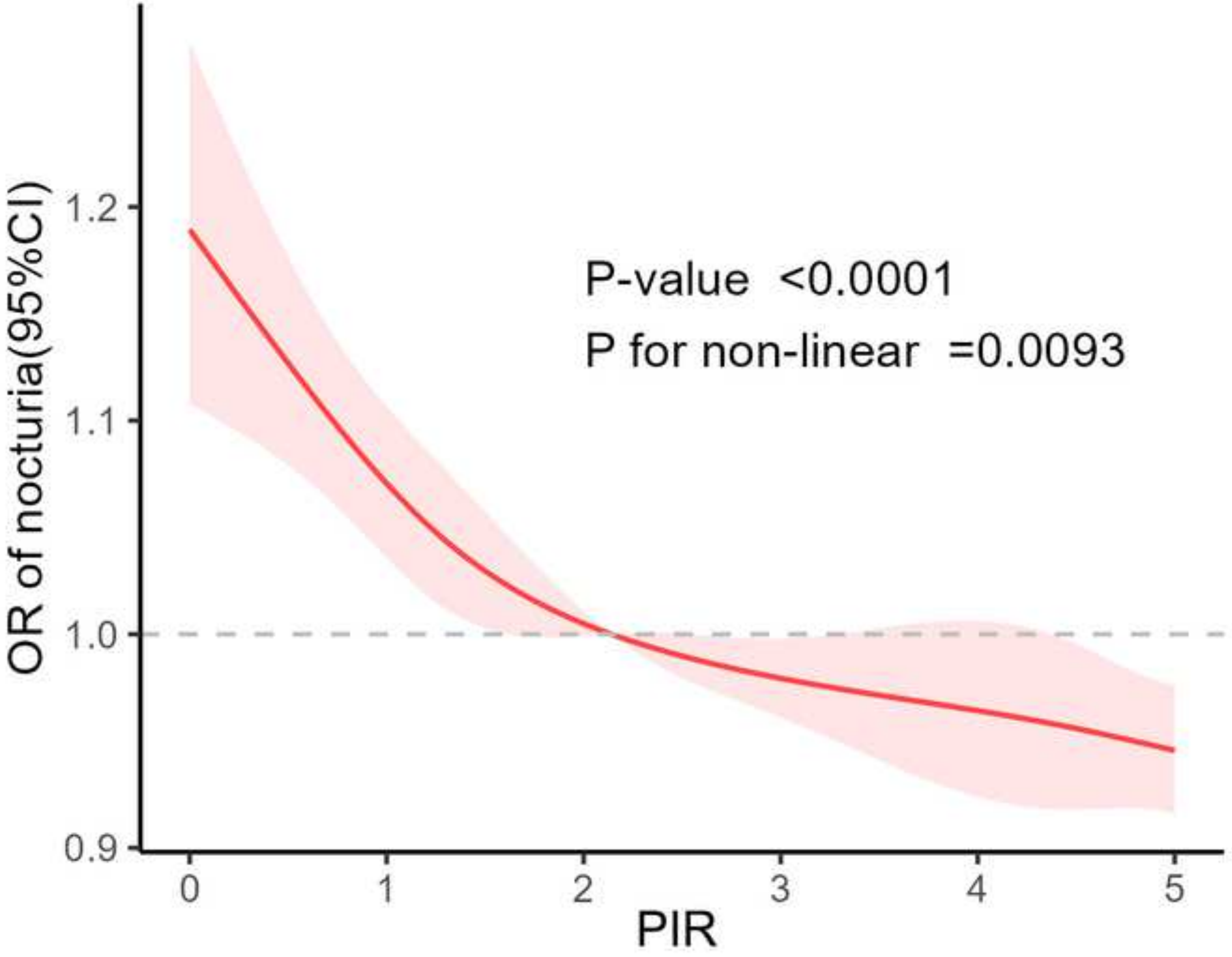

Figure 3 Click here to access/download;Figure;Figure 3.tiff

Characteristic	Total(N)	OR(95%CI)	P value	P for interaction
Gender				0.407
Male	3277	0.90(0.85 - 0.94)	<0.0001	
Female	3385	0.87(0.83 - 0.92)	<0.0001	
Age				0.179
20-34	1494	0.85(0.79 - 0.92)	0.007	
35-49	1590	0.85(0.80 - 0.91)	0.016	
50-64	1806	0.86(0.81 - 0.91)	<0.0001	
≥65	1772	0.99(0.92 - 1.06)	0.164	
Race				0.729
Mexican American	1224	0.95(0.87 - 1.04)	0.037	
Other Hispanic	537	0.93(0.83 - 1.04)	0.342	
Non-Hispanic White	3304	0.88(0.84 - 0.92)	<0.0001	
Non-Hispanic Black	1321	0.85(0.79 - 0.92)	<0.0001	
Other Race - Including Multi-Racial	276	0.84(0.72 - 0.97)	0.066	
Education				0.566
Less than high school	1954	0.89(0.83 - 0.96)	0.001	
High school	1590	0.83(0.77 - 0.90)	0.001	
More than high school	3118	0.90(0.86 - 0.95)	<0.0001	
Marital status				0.198
Widowed/Divorced/Separated/Never married	2568	0.85(0.80 - 0.90)	<0.0001	
Married/Living with partner	4094	0.90(0.86 - 0.94)	<0.0001	
Cotinine				0.193
<0.015	1304	0.96(0.89 - 1.03)	0.128	
0.015-3	3563	0.88(0.84 - 0.92)	<0.0001	
>3	1795	0.83(0.78 - 0.89)	<0.0001	
BMI				0.539
<25	1747	0.90(0.84 - 0.96)	0.002	
25-30	2224	0.86(0.81 - 0.91)	<0.0001	
≥30	2691	0.90(0.85 - 0.95)	0.005	
Drink at least 12 drinks/year				0.45
No	2002	0.87(0.82 - 0.93)	0.001	
Yes	4660	0.89(0.85 - 0.92)	<0.0001	
Hypertension history				0.66
No	2607	0.89(0.84 - 0.93)	0.002	
Yes	4055	0.88(0.84 - 0.92)	<0.0001	
Diabetes history				0.622
No	4766	0.88(0.84 - 0.92)	<0.0001	
Yes	1896	0.90(0.84 - 0.96)	0.007	

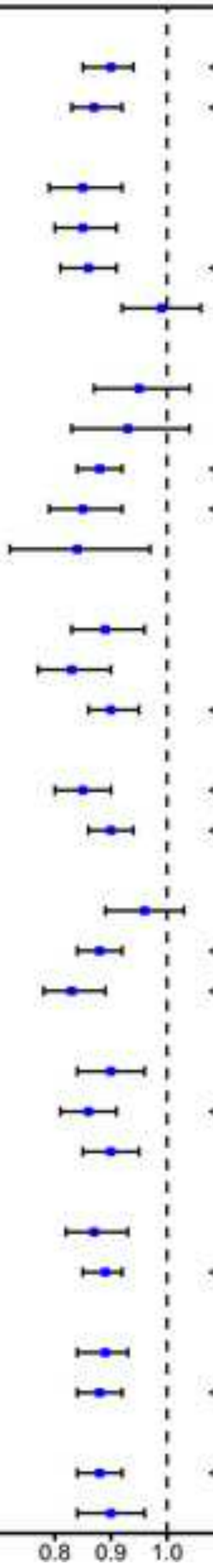

Supplement: Attachment — Submitted filename: PONE-D-24-06446_reviewer.pdf [file pone.0303927.s001.pdf]
