# Supplementary material for: Association between family income to poverty ratio and nocturia in adults aged 20 years and older: A study from NHANES 2005–2010
Source: PLoS One. 2024 May 20;19(5):e0303927. doi: 10.1371/journal.pone.0303927 (PMC11104595; doi:10.1371/journal.pone.0303927)
Supplement: Attachment — Submitted filename: Response to Reviewers.docx [file pone.0303927.s002.docx]

We would like to sincerely thank the editor and reviewers for their thorough evaluation of my manuscript. Their valuable feedback and constructive criticism have played a crucial role in refining the quality of the article. I am grateful for their dedicated efforts during the review process and for taking their comments seriously and making responsible revisions to my work.

**Review Comments**

**Reviewer 1:**

Question 1：This article discusses an important topic in the field of public health. It is well written. The data was appropriately analyzed and results were well presented and discussed. Relevant literature was covered.

**Our** response：We have noted that reviewer 1 has not put forward any questions that need to be revised. We sincerely appreciate the thorough review and acknowledgment of my paper by the first reviewer.

**Reviewer 2**

Question **1:** Please provide more details on the specific NHANES survey cycles used and the handling of missing data.

**Our response:** We listed three specific NHANES cycles used in the literature (line 89) and conducted a detailed statistical analysis of missing data (Figure 1).

Question **2:** The rationale for the categorization of PIR into three groups (PIR <1, PIR 1-4, PIR > 4) should be better explained and supported by relevant literature or guidelines.

**Our response:** We classified PIR based on the following references (DOI: 10.3389/fpubh.2022.873805, 10.3389/fendo.2023.1160625 and 10.3389/fonc.2023.1265356). Additionally, eligibility for subsidies under the Patient Protection and Affordable Care Act was used to assess the middle- and high-income groups.

Question **3：**The authors should consider discussing the potential clinical implications of their findings, such as the potential use of PIR as a risk marker for nocturia or the importance of addressing socioeconomic disparities in the management of nocturia.

**Our response:** We further explored the potential utility of PIR as a risk marker for nocturia (line 227-234) and the significance of socioeconomic disparities in managing nocturia (line 206-210).

**Reviewer 3**

Question **1:** A modification in the title to include the age range utilized in the study.

**Our response:** We revised the title to "Association between family income to poverty ratio and nocturia in adults aged 20 years and older: a study from NHANES 2005-2010" using " in adults aged 20 years and older " to denote the age range (line 2).

Question **2:** Further discussion on potential mechanisms underlying the observed correlation and addressing limitations such as the cross-sectional nature of the study and the need for additional prospective research.

**Our response:** We further discussed the potential mechanisms underlying the correlation between nocturia and PIR, analyzing its practical application value in clinical relevance (line (line 227-234). Finally, we proposed the need for additional prospective and longitudinal studies to validate our experiments' conclusions and overcome our research's limitations (line 253-256).

Question **3:** Clarification on potential biases and their mitigation strategies would enhance the manuscript's robustness.

**Our response:** We further explored the possibility of bias. Mitigation strategies such as conducting further longitudinal and prospective studies and more extensive scale studies are also proposed (line245-256).

**Editorial Formatting Comments**

Question **1:** Include the number and proportion of the sample with nocturia.

**Our response:** In the results section of the abstract, we included information regarding the number and proportion of the sample with nocturia (line 38-42).

Question **2:** Include the test statistics.

**Our response:** In the results section of the abstract, we added the p-value of the correlation between PIR and nocturia in Model 3 (line 44).

Question **3：**The conclusion of the abstract could be improved by making it more concise and focused on the key findings and implications of the study.

**Our response：**We revised the conclusion section of the abstract based on the feedback provided in the attachment (line 48-52).

Question **4:** Explain the rationale for using three models.

**Our response:** Model 1 was left unadjusted to observe the nocturia and PIR relationship visually. Model 2 included some demographic and socioeconomic factors as confounding variables. Model 3 included all possible confounding variables to resemble the patient's situation closely. Therefore, using three models allows for progressive correlation analysis between nocturia and PIR, making our experimental design more logical.

Question **5:** Ensure uniformity in the fonts.

**Our response:** We sincerely apologize for the error. We have revised the table to ensure consistency in the text (Table 1). Additionally, we have modified the affiliations to make them more formal (line 7, line 9-12).
